# A Multi-System Approach Assessing the Interaction of Anticonvulsants with P-gp

**DOI:** 10.1371/journal.pone.0064854

**Published:** 2013-05-31

**Authors:** David Dickens, Siti R. Yusof, N. Joan Abbott, Babette Weksler, Ignacio A. Romero, Pierre-Olivier Couraud, Ana Alfirevic, Munir Pirmohamed, Andrew Owen

**Affiliations:** 1 Department of Molecular and Clinical Pharmacology, University of Liverpool, Liverpool, United Kingdom; 2 King's College London, Blood–Brain Barrier Group, Institute of Pharmaceutical Science, London, United Kingdom; 3 Weill Cornell Medical College, New York, New York, United States of America; 4 Department of Life Sciences, The Open University, Milton Keynes, United Kingdom; 5 INSERM, Paris, France; 6 CNRS, Paris, France; 7 Univ Paris Descartes, Paris, France; Universidad de Castilla-La Mancha, Spain

## Abstract

30% of epilepsy patients receiving antiepileptic drugs (AEDs) are not fully controlled by therapy. The drug transporter hypothesis for refractory epilepsy proposes that P-gp is over expressed at the epileptic focus with a role of P-gp in extruding AEDs from the brain. However, there is controversy regarding whether all AEDs are substrates for this transporter. Our aim was to investigate transport of phenytoin, lamotrigine and carbamazepine by using seven *in-vitro* transport models. Uptake assays in CEM/VBL cell lines, oocytes expressing human P-gp and an immortalised human brain endothelial cell line (hCMEC/D3) were carried out. Concentration equilibrium transport assays were performed in Caco-2, MDCKII ±P-gp and LLC-PK1±P-gp in the absence or presence of tariquidar, an inhibitor of P-gp. Finally, primary porcine brain endothelial cells were used to determine the apparent permeability (P_app_) of the three AEDs in the absence or presence of P-gp inhibitors. We detected weak transport of phenytoin in two of the transport systems (MDCK and LLC-PK1 cells transfected with human P-gp) but not in the remaining five. No P-gp interaction was observed for lamotrigine or carbamazepine in any of the seven validated *in-vitro* transport models. Neither lamotrigine nor carbamazepine was a substrate for P-gp in any of the model systems tested. Our data suggest that P-gp is unlikely to contribute to the pathogenesis of refractory epilepsy through transport of carbamazepine or lamotrigine.

## Introduction

Epilepsy affects 50 million people worldwide. Antiepileptic drug (AED) therapy is ineffective in approximately 30% of patients, who continue to have seizures despite regular dosing [1]. The mechanisms underlying drug resistance are not fully understood. The transport hypothesis proposes that over-expression of P-gp (P-glycoprotein, ABCB1, MDR1) at the blood-brain barrier (BBB) may increase drug efflux and limit access of AEDs to the epileptic focus [2]. The key finding of high levels of P-gp expression in temporal lobe specimens has supported its role in the pathogenesis of refractory epilepsy [3]. This has led to the hypothesis that up-regulation of P-gp at the epileptic focus of refractory epilepsy patients plays a causal role in the lack of drug response by reducing the concentrations of AEDs at the epileptic focus. This is an active area of clinical research as a possible target for treatment of refractory epilepsy. At least four clinical trials are active in North America alone studying either P-gp activity in epilepsy by PET scan studies or testing P-gp inhibition as an adjuvant treatment for refractory epilepsy (http://www.clinicaltrials.gov/). However the hypothesis that P-gp has a causal role in refractory epilepsy remains unproven, but recent data have confirmed that P-gp protein expression is up-regulated at the epileptic focus in refractory patients [4].

P-gp has a wide anatomical distribution and plays an important role in the bioavailability and disposition of many xenobiotics, as shown using *MDR1* knockout mice [5]. P-gp is expressed in the kidney, liver, blood–brain barrier, and intestine and can act as a biological “gatekeeper”, limiting the accumulation into sensitive tissues such as brain, testis and lymphocytes [6]. P-gp inhibition has been suggested as an adjuvant therapy to alter the bioavailability and distribution of substrate drugs into tissues such as the brain and peripheral tissues. Inhibitors can be categorised into three groups: 1^st^ generation transport inhibitors are licensed drugs (e.g. cyclosporine), shown to be high affinity substrates; 2^nd^ generation inhibitors (e.g. PSC-833), which are more specific for P-gp; and 3^rd^ generation inhibitors (e.g. tariquidar), which show the highest specificity for P-gp. However, there are continuing contradictory reports on whether AEDs are substrates for P-gp and this is summarised by two recent reviews [7], [8]. The use of *MDR1* knockout mice has shown that rodent P-gp affects brain accumulation of phenytoin but not lamotrigine or carbamazepine [9], [10], [11], [12]. Additionally, bi-directional transport assays utilising Caco-2 cells and cells that are stably transfected with human P-gp (MDCKII) have found no P-gp interaction for phenytoin, lamotrigine or carbamazepine [12], [13]. Many studies have shown differences in affinity of compounds between human and mouse P-gp [14], [15]. Therefore, previous discrepancies may be explained by differences in affinity for human and rodent P-gp [16]. Porcine P-gp demonstrates a high homology (90.8%) to human P-gp [17] and therefore porcine cells may be a more appropriate model. Using a modification in transporter assay design (equilibrium method) most AEDs (except carbamazepine) were found to be weak substrates of human P-gp in the LLC-PK1-Pgp transfected cell line [18]. One study conducted in Caucasians also showed an association of *ABCB1* polymorphisms with refractory epilepsy [19], while a larger mixed international cohort found no association with *ABCB1* genotype [20]. A subsequent meta-analysis found no association between *ABCB1* SNPs and refractory epilepsy [21].

There are many diverse assays to determine if a compound is a P-gp substrate with a recent review proposing a decision tree for identifying P-gp substrates [22]. Bi-directional transporter assays utilising Caco-2 or P-gp over-expressing polarized epithelial cells were proposed as optimal, with a net flux ratio >2 and a P-gp inhibitor reducing the transport, being indicative of a positive response [22]. Another study assessed three *in-vitro* systems for characterisation of P-gp substrates and found the bidirectional assays to be the method of choice, while the calcein AM and P-gp ATPase assays were also useful in characterising P-gp substrates into different categories [23]. This emphasises the need to use multiple transporter assay systems for testing of potential substrates.

Given the conflicting evidence regarding P-gp-mediated transport of AEDs, the aim of the present work was to use multiple assay systems to investigate whether phenytoin, lamotrigine and carbamazepine interact with P-gp. We present data on these 3 drugs in seven *in-vitro* transport systems.

## Materials and Methods

### Materials

Tariquidar was synthesised by Dr. Oliver Langer, Medical University of Vienna, Department of Clinical Pharmacology, Austria. ^3^H-digoxin and ^14^C-phenytoin were purchased from PerkinElmer (Beaconsfield, UK) with specific activity of 23.5Ci/mmol and 53.1mCi/mmol respectfully. ^14^C-carbamazepine was provided by Ciba (Basel, Switzerland) with specific activity of 49mCi/mmol and ^14^C-lamotrigine was a kind gift from GSK (Stevenage, U.K.) with specific activity of 52.9mCi/mmol. All other chemicals and reagents were purchased from Sigma-Aldrich unless otherwise stated.

### Cell Culture

Caco-2 cells, MDCKII±Pgp, hCMEC/D3 cells, CEM and VBL_100_ cells were grown as previously described [24], [25], [26]. LLC-PK1 cell line transfected with human *ABCB1* and respective wildtype LLC-PK1 cells were kindly provided by Prof. Borst (NKI, Netherlands) [27] and were cultured in Medium 199 and 10% FCS.

The porcine brain microvessel isolation and culture protocol was based on previously published papers [28], [29]with modifications [30]. Briefly, brains from six pigs (Cheale Meats Ltd., Brentwood, UK) were washed. The grey matter was homogenised, filtered, digested, centrifuged and microvessels cryopreserved. Porcine brain endothelial cells (PBECs) were cultured on rat-tail collagen (330µg/ml)/fibronectin (7.5µg/ml)-coated plastic-ware in DMEM supplemented with 10% bovine plasma derived serum (First Link, UK), heparin (125µg/ml), L-glutamine (2mM), penicillin/streptomycin (100U/100µg/mL) and puromycin (4µg/mL);

### *X. laevis* oocyte Isolation and Microinjection with cRNA

*Xenopus laevis* oocyte expression plasmid (pBluescriptII-KSM) containing either wild-type human *ABCB1* cDNA or an ATPase deficient mutant of *ABCB1* (G1601A, G1602T) were utilised. The ATPase deficient mutant encodes for a transporter with an amino acid change (G534D) that has previously been ectopically expressed and shown to be expressed at equivalent levels as the wild type protein and with normal membrane insertion but has a complete loss of drug-stimulated ATPase activity [31]. To generate cRNA, the T3 mMessage *in-vitro* transcription kit was utilised following manufacturer’s instructions. As described previously, oocytes were extracted, injected with the cRNA or distilled water (50nl for each) and were maintained for 3 days [32].

### Cellular Accumulation Assay in hCMEC/D3, CEM and VBL_100_ Cells

Cells were equilibrated in transport buffer (HBSS containing 25mM HEPES and 0.1% (w/v) bovine serum albumin at pH 7.4) at 37°C with accumulation assays performed as previously described [24], [25]. Studies of drug transport utilised a tracer concentration of radiolabelled compound (0.3µCi/ml) with sufficient non-radiolabelled compound added to give a final concentration of drug.

### Concentration Equilibrium Assay in Caco-2, LLC-PK1 and MDCKII Cells

Caco-2 cells were seeded onto transwell polycarbonate membrane inserts (Corning) with pore size 0.4 µm and membrane diameter of 24 mm and cultured for 21 days. MDCKII±P-gp and LLC-PK1±P-gp were seeded and cultured for 8 days. The minimum trans-endothelial electrical resistance (TEER) acceptable for each cell line was above 100 Ω.cm^2^. For transport experiments, cell monolayers were equilibrated in transport buffer and replaced with appropriate solutions of 1.5ml in the apical and 2.6ml in the basal compartment.

### *X. laevis* Oocyte P-gp Drug Transport Assay

For oocyte transport studies involving measurement of efflux, oocytes were injected with a radiolabelled drug in the absence or presence of an P-gp inhibitor to give final intra-oocyte concentrations of 1µM for digoxin or 5µM for phenytoin. An intra-oocyte volume of 500nl was used to calculate the final concentrations [33]. The injected oocytes were added to transport buffer in the presence of the appropriate concentration of inhibitor and the efflux from each individual oocyte was determined after 45 minutes at 22°C. For accumulation, oocytes were incubated in transport buffer containing 20µM drug in the presence or absence of inhibitors for 1 hour at 22°C. After incubation, the oocytes were transferred to ice-cold HBSS, washed three times with individual oocytes solubilised in 10% SDS.

### Permeability Assay Utilising PBECs

The PBECs were seeded onto rat-tail collagen/fibronectin-coated transwell filter inserts at a density of 1×10^5^cells/cm^2^. At confluence, culture medium was replaced by serum-free medium containing hydrocortisone (550nM), and 8-4-chlorophenylthio-cAMP (250µM) and RO-20-1724 (17.5µM) for 24 hours. This was conducted to elevate intracellular cAMP and thereby encourage differentiation of BBB phenotype including tight junctions. Cell monolayers were used when TEER exceeded 340 Ω.cm^2^. For permeability assays, DMEM without phenol red with added HEPES (25mM) and 0.1% BSA was pH adjusted to 7.4 and used as transport medium. Cells were incubated at 37°C for 1 hour with radiolabelled compounds and cold compound to give a final concentration of 6µM in the apical compartment for apical-to-basal direction of transport. Samples were taken from both compartments.Apparent permeability (Papp) in cm/s for the compounds studied was calculated as previously described [30].

### Analysis of P-gp Expression

Flow cytometric analysis of cell surface P-gp expression was carried out in all cell lines as described previously [34] using the monoclonal antibody UIC2 that binds to an extracellular epitope of P-gp. The median fluorescence was determined and normalised against that of a low P-gp expressing cell line (CEM) to produce a relative fluorescent unit (RFU).

### Statistical Analysis

All data are presented as mean ± standard deviation. To assess statistical significance the samples were compared using a *t-*test or by ANOVA followed by Tukey-Kramer test for multiple comparisons using GraphPad Prism 5. The significance values were as follows: * P<0.05, ** P<0.01, *** P<0.001.

## Results

### Protein Expression of P-gp in Transport Models

**Figure 1:**
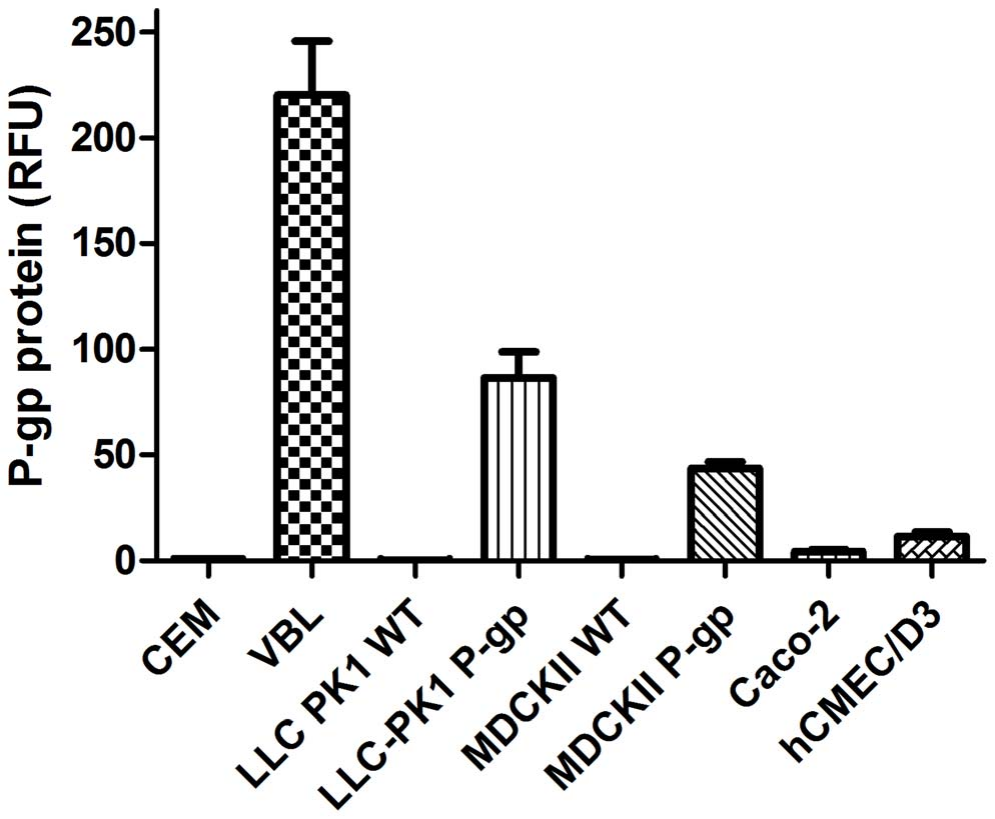
Relative membrane expression of P-gp in cell lines. Expression of P-gp determined using an extracellular epitope-specific antibody with flow cytometry performed. P-gp protein is expressed as relative fluorescence units with values relative to the CEM cell line and expressed as mean ±SD (n = 3).

The membrane protein expression of P-gp was determined in the cells using an epitope specific antibody to P-gp with relative fluorescence units compared to the low P-gp expressing CEM cells (Fig. 1). The VBL_100_ cells were found to have 220 times more membrane P-gp expression than the CEM parental cells (used as baseline). The transfected LCC-PK1-P-gp and MDCK-P-gp cells had P-gp expression 86 and 43 times the baseline value, respectively. Endogenous expression of P-gp in Caco-2 and hCMEC/D3 cells was found to be 4.2 and 11.6 times the expression in CEM cells.

### Model of Multi-drug Resistance: Accumulation into CEM and VBL_100_ Cells

**Figure 2:**
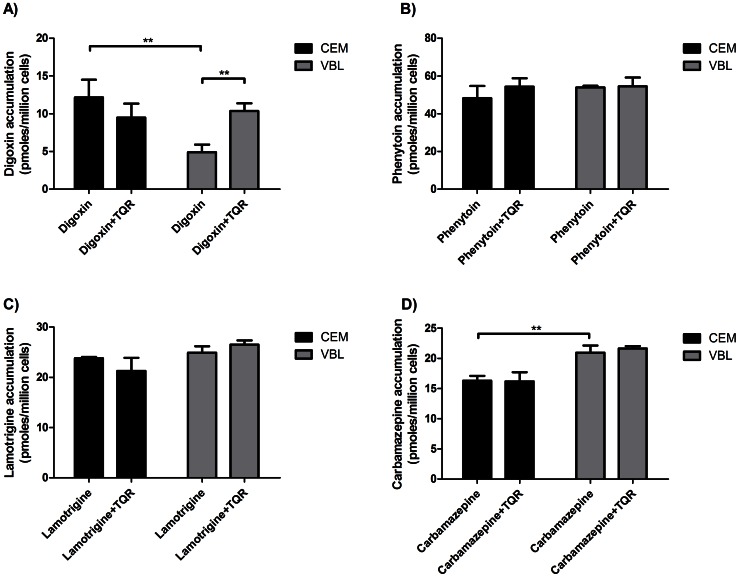
Uptake of AEDs into CEM and VBL_100_ cell lines. Cells were incubated for 30 minutes in transport buffer with (a) 5µM ^3^H-digoxin or (b) 5µM ^14^C-phenytoin or (c) 5µM ^14^C-lamotrigine or (d) 5µM ^14^C-carbamazepine in the absence or presence of 300nM tariquidar (TQR). Uptake into cell lines is shown as pmoles per million cells and the data is expressed as mean ±SD (n = 3). ** Significantly different from the appropriate control sample as indicated (P<0.01).

To determine whether phenytoin, lamotrigine and carbamazepine were P-gp substrates, transport studies in an *in-vitro* model of multi-drug resistance were performed. The VBL_100_ lymphoblastoid cell line has been generated by continuous culture of CEM cells with vinblastine, which has induced multi-drug resistance through over-expression of P-gp [35]. Accumulation studies with the drugs in the T-cell leukaemia lines CEM and VBL_100_ cells were assessed in the presence or absence of tariquidar. Tariquidar is a non-competitive inhibitor of P-gp that can inhibit both P-gp and BCRP. The increased expression of P-gp in the drug-resistant VBL_100_ cells resulted in less accumulation of digoxin (used as a positive control) than in CEM cells, while tariquidar restored accumulation (Fig. 2a), confirming that cellular concentrations of digoxin were dependent on functional P-gp activity. No significant decrease in accumulation or tariquidar inhibition was observed for phenytoin, carbamazepine and lamotrigine suggesting that P-gp does not contribute to accumulation of these three drugs (Fig. 2b,c,d) in this model. A significantly higher accumulation of carbamazepine was observed in VBL_100_ than in CEM cells but this was not inhibited by tariquidar suggesting the involvement of an as yet unidentified influx transporter in these cells.

### Transcellular Permeability: Concentration Equilibrium Assay in Caco-2, LLC-PK1-ABCB1 and MDCKII-ABCB1 Cells

Caco-2 cells expressing endogenous levels of P-gp were used in the concentration equilibrium transport assay with phenytoin, lamotrigine and carbamazepine. The concentration equilibrium approach has been suggested to be a sensitive assay for transcellular permeability studies [18]. As a positive control to show functional P-gp, digoxin was shown to be transported into the apical compartment and this was inhibited by tariquidar (Fig. S1a,b). No polarised transport of phenytoin, lamotrigine or carbamazepine was observed in the Caco-2 cells into either compartment over a 4 hour time course (Fig. S1c,d,e).

**Figure 3:**
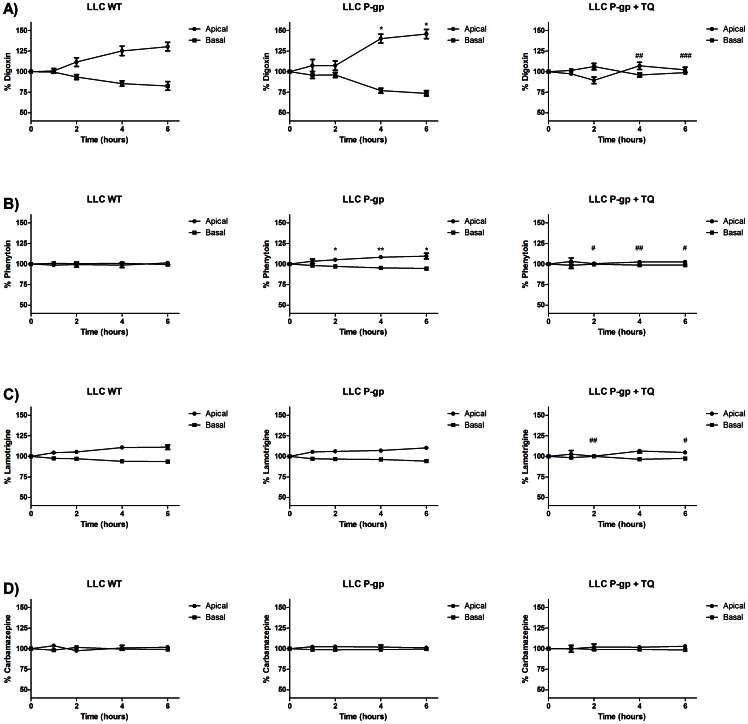
Concentration equilibrium approach in LLC-PK1 transfected with human P-gp for the transport of AEDs. Transport of a) 5µM ^3^H-digoxin or (b) 5µM ^14^C-phenytoin or (c) 5µM ^14^C-lamotrigine or (d) 5µM ^14^C-carbamazepine in LLC-PK1±P-gp in the absence or presence of 300nM tariquidar. Samples were taken at each indicated time point over a 6 hour time course with the percentage concentration of drug determined in the apical and basal compartments. Data are expressed as mean ±SD (n = 3). * significantly different compared to wild type cells (* P<0.05, ** P<0.01). # significantly different compared to LLC-PK1+P-gp cells in the absence of tariquidar (# P<0.05, ## P<0.01, ### P<0.001).

Since P-gp expression in Caco-2 cells is at an endogenous level, it is possible that the concentration equilibrium approach may give a negative result due to the relatively low expression of P-gp. Therefore a cell line stably transfected with human P-gp was used. LLC-PK1-P-gp cells showed significantly higher transport of digoxin into the apical compartment compared to the parental LLC-PK1 cells; this was inhibited by tariquidar confirming functional P-gp activity (Fig. 3a). Transport of phenytoin into the apical compartment was also higher in LLC-PK1-P-gp cells and inhibitable by tariquidar, showing that at high expression levels, P-gp-mediated phenytoin transport can be demonstrated (Fig. 3b). Lamotrigine transport was observed into the apical compartment in both LLC-PK1-P-gp and the parental line but was only partly inhibited by tariquidar (Fig. 3c). Since the transport profile was the same in control cells as in the transfected cells, it is highly unlikely that this is mediated by P-gp and suggests the involvement of an unidentified drug transporter. To ensure this was not concentration-dependent, two additional concentrations were tested and the same transport profile was observed for 20µM and 40µM compared to 5µM (Fig. S2a,b).

To validate the result observed in the LLC-PK1 expressing P-gp cells in an additional independent expression system, MDCKII cells expressing P-gp were used. A significant increase in apical concentration of digoxin was observed in the P-gp expressing MDCKII cells compared to the parental cells. Tariquidar significantly reducing the apical concentration increase in the cells showing functional P-gp activity (Fig. S3a). Similar to the LLC-PK1-P-gp cells more phenytoin was transported into the apical compartment in the MDCKII- P-gp cells compared to the parental MDCKII cells and this was inhibited by tariquidar (Fig. S3b). Lamotrigine transport over the 6 hour time course was similar in both the MDCKII cell lines (Fig. S3c). Similarly, no difference was observed for the transport of carbamazepine (Fig. S3d).

### Accumulation in X. *laevis* oocytes Expressing P-gp

**Figure 4:**
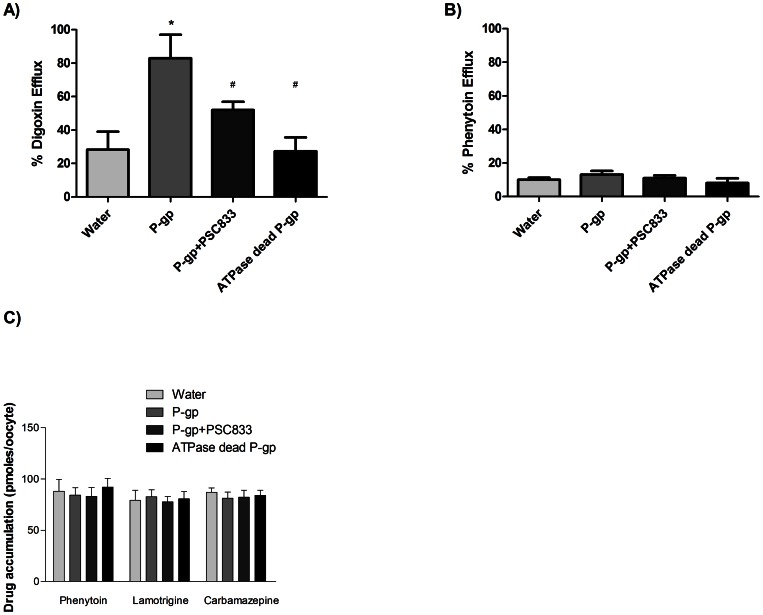
Transport of AEDs in oocytes expressing human P-gp. a) Percentage efflux of digoxin from cRNA injected oocytes compared to water injected negative control oocytes. Intra-oocyte concentration of 1µM ^3^H-digoxin ±40µM PSC833 with data expressed as mean ± SD (n≥3, 8–10 oocytes per experiment). The significance values are * (P<0.05) compared to water injected oocytes and # (P<0.05) compared to P-gp injected oocytes. b) Percentage efflux of ^14^C-phenytoin from cRNA injected oocytes compared to water injected negative control oocytes. Intra-oocyte concentration of 5µM ^14^C-phenytoin ±40µM PSC833 with data expressed as mean ± SD (n≥3, 8–10 oocytes per experiment). c) Accumulation of ^14^C-phenytoin, ^14^C-lamotrigine or ^14^C-carbamazepine, in oocytes expressing human P-gp. The accumulation of drug into oocytes with 20µM drug ±40µM PSC833 in transport buffer was determined as pmoles per oocyte, from oocytes expressing human wild-type P-gp, triple SNP variant or ATPase dead mutant (AD) compared to water injected negative control oocytes. Data are expressed as mean ± SD (n≥3, 8–10 oocytes per experiment).

Since mammalian cell lines express multiple transporters other than just P-gp, a *Xenopus laevis* expression system was utilised (Fig. 4) as *Xenopus laevis* oocytes express very few endogenous transporters compared to mammalian cells [36]. Digoxin was used as a positive control and showed higher efflux from P-gp injected oocytes than from water-injected oocytes. This was also dependent on the ATPase activity of P-gp and was reduced by the P-gp inhibitor, PSC-833 (Fig. 4a). No difference in phenytoin efflux was observed in P-gp -expressing oocytes compared to the water-injected oocytes (Fig. 4b). Uptake into oocytes was also studied for phenytoin, lamotrigine or carbamazepine. No differences in phenytoin, lamotrigine or carbamazepine accumulation were detected in the oocytes suggesting that no P-gp-mediated transport occurred (Fig. 4c).

### Transport in Human Brain Endothelial Cells

**Figure 5:**
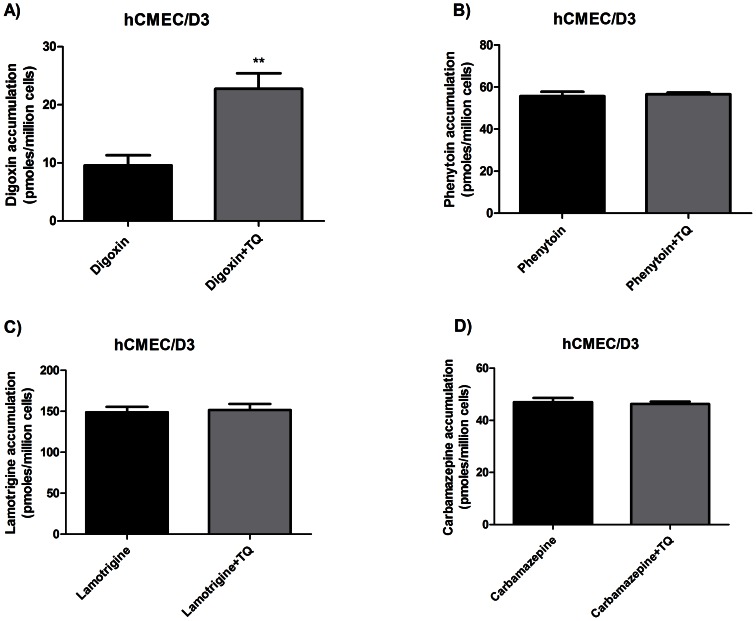
Uptake of AEDs into a human brain endothelial cell line (hCMEC/D3). Cells were incubated for 30 minutes in transport buffer with (a) 5µM ^3^H-digoxin or (b) 5µM ^14^C-phenytoin or (c) 5µM ^14^C-lamotrigine or (d) 5µM ^14^C-carbamazepine in the absence or presence of 300nM tariquidar (TQR). Uptake into cell lines shown as pmoles per million cells and the data is expressed as mean ±SD (n = 3). ** significantly different compared to cells without inhibitor (** P<0.01).

To determine whether P-gp mediated transport of the three AEDs occurred in human brain endothelial cells, hCMEC/D3 cells were utilised. hCEMC/D3 cells are an immortalised human brain endothelial cell line with endogenous expression of P-gp [37], and represent an *in vitro* model of the human BBB. Functional activity of P-gp was shown by uptake studies where the P-gp inhibitor tariquidar enhanced the accumulation of digoxin (Fig. 5a). No effect of tariquidar was seen on accumulation of phenytoin, carbamazepine or lamotrigine suggesting no P-gp -mediated transport of these AEDs in this system (Fig. 5b,c,d).

### Transcellular Transport in Primary Porcine Brain Endothelial Cells

**Figure 6:**
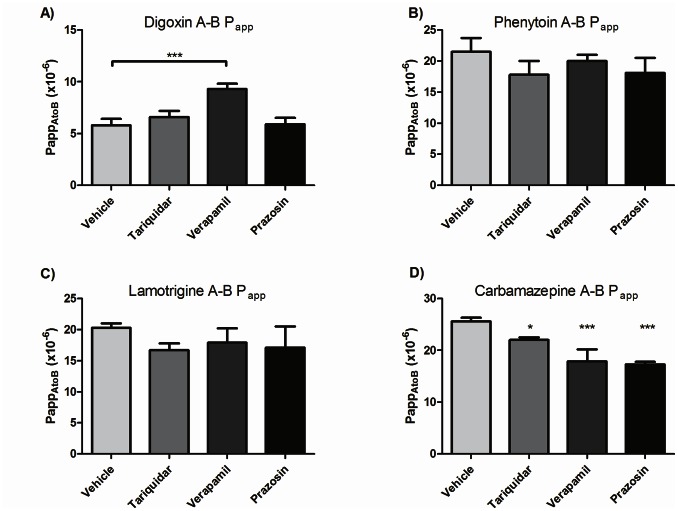
Apparent permeability of AEDs in apical to basal direction in a primary porcine brain endothelial monolayer. Cells were grown on transwells and drug added to apical compartment in transport buffer with (a) 6µM ^3^H-digoxin or (b) 6µM ^14^C-phenytoin or (c) 6µM ^14^C-lamotrigine or (d) 6µM ^14^C-carbamazepine in the absence or presence of 300nM tariquidar, 100µM verapamil and 100µM prazosin. Data are expressed as mean ±SD (n = 3) with * indicating significant difference compared to control P_app_s (* P<0.05, ** P<0.01, *** P<0.001).

Primary porcine brain endothelial cells were used to investigate the apical to basolateral transport of AEDs in a two compartment transwell. Digoxin was used as a positive control drug and verapamil inhibition increased the digoxin P_app_ (Fig. 6a). However, the more specific inhibitor, tariquidar, did not affect the permeability (Fig. 6a). The P_app_ for phenytoin and lamotrigine was not affected by verapamil or tariquidar treatment while for carbamazepine transport a decrease in apical to basolateral P_app_ was observed (Fig. 6b,c,d). This active transport of carbamazepine was unlikely to be mediated by P-pg, because the location of this transporter at the apical membrane of brain endothelial cells [38] is expected to mediate only the basolateral to apical direction of transport.

## Discussion

The role for P-gp in refractory epilepsy remains controversial and there is discordant data with respect to the clinical significance and transport of AEDs [7], [8]. However an active clinical program is both investigating P-gp inhibitors as adjuvant therapy and utilising PET scanning to assess P-gp activity in patients. The field requires additional evidence to substantiate the role of P-gp in refractory epilepsy, particularly regarding whether P-gp mediates AED transport (Table 1). The present study represents a multi-system approach to investigate whether phenytoin, lamotrigine or carbamazepine interact with P-gp (Table 2). Models of multidrug resistance, specific over-expression systems and cells expressing P-gp at physiologically relevant densities were used. Lamotrigine and carbamazepine showed no P-gp interaction in any of the seven transport systems studied, suggesting they are not substrates and thus their cellular disposition is unlikely to be affected by P-gp expression levels or activity.

**Table 1:** Summary of the published evidence for phenytoin, lamotrigine and carbamazepine being substrates of P-gp.

Transport System		Phenytoin	Lamotrigine	Carbamazepine
**MDR1 KO Mouse** [10], [11], [12], [34]		Yes/No	No	No
**Transfected mouse P-gp cell line;** **Bi-directional transwell method** [12], [16]		Yes/No	No	No
**Transfected human P-gp cell lines** [12], [16], [18]	**Bi-directional transwell**	No	No	No
	**Equilibrium**	Yes	Yes	No
**Caco-2 Bi-directional transwell** [11], [13]		No	No	No
**Primary human brain endothelial cells** [45]		No	ND	ND
**Primary epileptic derived human brain endothelial cells** [45]		Yes	ND	ND
**Microdialysis/brain access in rodent models** [9], [51], [52], [53]		Yes	Yes	Yes
**Stimulation of P-gp ATPase activity** [12]		No	No	No
**Calcein-AM P-gp inhibtion assay** [12]		No	No	No

The P-gp inhibitors included in the table are the blockers that were positive for the specific technique or cell type. ND: investigation not done.

**Table 2:** Summary of the interactions with P-gp observed in the present multi-model study.

Transport System	PHT	LTG	CBZ	Advantages	Disadvantages
**CEM/VBL cells**	No	No	No	Very high expressionof P-gp	Intracellular, other transporters
**Caco-2 (equilibrium method)**	No	No	No	High endogenousexpression of P-gp	Specificity
**MDCK±P-gp (equilibrium method)**	Yes –“weak”	No	No	High over-expressionof P-gp	Endogenous canine transporters
**LLC-PK1±P-gp (equilibrium method)**	Yes –“weak”	No	No	High over-expressionof P-gp	Endogenous porcine transporters
**Human brain endothelial cell line (hCMEC/D3)**	No	No	No	High endogenousexpression of P-gp	Intracellular, specificity
**Primary porcine brain endothelial**	No	No	No	Primary cells	Specificity
***Xenopus laevis*** ** oocytes expressing human P-gp**	No	No	No	Specificity	Non-mammalian physiology

CEM cells have very low P-gp expression and together with VBL_100_ cells, which have a very high P-gp expression, are a model for drug resistance. Transport of digoxin and high P-gp protein expression was shown in in the VBL_100_ cells. However, no transport of AEDs by P-gp in this model was observed. It should be noted that other drug transporters including influx transporters [39] differ between CEM and VBL_100_ cells but despite this, they have been successfully used to identify P-gp substrates such as imatinib and ABT-263 [24], [40].

No transport of phenytoin, carbamazepine and lamotrigine by human P-gp was previously shown by a conventional bidirectional transcellular permeability method in three cell lines that express P-gp; Caco-2, LLC-PK1- P-gp and MDCKII- P-gp [12], [13], [16]. However, recently a concentration equilibrium approach was proposed to be more sensitive than the conventional method [18], [41]. In Caco-2 cells that express P-gp at endogenous levels, this assay proved to be negative for all three AEDs tested. It is possible that expression of P-gp in Caco-2 cells is not high enough to detect the transport of weak substrates. Indeed, LLC-PK1 cells transfected with P-gp did show transport of phenytoin by P-gp that was inhibited by tariquidar. However, no transport of lamotrigine or carbamazepine was observed. MDCKII cells transfected with human P-gp were also used to validate these findings. A previously published paper found that phenytoin and lamotrigine were transported by P-gp in LLC-PK1-P-gp by the concentration equilibrium approach [18] but in the present study, lamotrigine was not a substrate in these cells across a range of concentrations. Luna-Tortos *et al* observed minor P-gp mediated transport of lamotrigine, but this was not fully inhibited by tariquidar (only 60%) implying the possible involvement of other endogenous transporters. Interestingly, a recent study utilising the concentration equilibrium approach proposed that P-gp does not transport carbamazepine, but does transport its active metabolite carbamazepine-10,11-epoxide [42]. The metabolites of anticonvulsants could be the focus of future studies to investigate the possibility that they are substrates of P-gp. The suggested advantage of the concentration equilibrium approach is that it might reduce the passive permeability component. However, it should be noted that the concentration of an actively transported drug is highly unlikely to be identical on both sides of a biological barrier *in vivo*.

The *Xenopus laevis* oocyte expression system was validated for P-gp protein expression (data not shown) and transport function. This expression system has several advantages for drug transport due to the transient nature of protein expression and low expression of endogenous transporters in oocytes [36]. No P-gp mediated transport of the three AEDs was observed in this model. If phenytoin is a weak substrate for P-gp this might explain why it was not identified as a substrate using this approach. However, it is important to note that two well-recognised P-gp substrates, digoxin (Fig. 4A) and imatinib (data not shown) were confirmed to be substrates in this system.

hCMEC/D3 cells are an immortalised human brain endothelial cell line [43]. No inhibition of transport of the three AEDs by P-gp inhibitors was observed even though high protein expression and inhibition of transport of a model substrate was observed. As immortalised cells can lose important characteristics, primary porcine brain endothelial cells were also used. PBMEC retain many barrier characteristics and transport pathways of the *in-vivo* BBB [44]. We have recently validated a PBMEC model that has both P-gp expression and activity [30]. Transcellular permeability studies with these cells showed transport of digoxin in the apical to basal direction which was increased by verapamil, but no corresponding increase was observed for the three AEDs. hCMEC/D3 and porcine brain endothelial cells are both representative of normal brain endothelial cells in terms of protein density of transporters. It should be noted that in a previous study, primary epileptic human brain endothelial cells (but not the control primary cells) were shown to transport phenytoin, which was inhibited by tariquidar [45].

A potential issue to consider when investigating interactions with P-gp is that the concentrations tested might be near to or above the IC_50_ for P-gp inhibition and this might then mask active transport. The three AEDs tested in this study have been described as either non- P-gp inhibitors or weak inhibitors. The concentrations tested are lower than any P-gp inhibitory effect and would therefore be unlikely to yield a false negative result. For example in LLC-PK1-P-gp cells, drug treatment with 100µM of either phenytoin, carbamazepine or lamotrigine showed no inhibition of the uptake of the P-gp substrate [46]. Only carbamazepine at 100µM (but not at 10µM) was able to inhibit uptake of calcein AM into porcine brain endothelial cells [46].

Another variable to consider is the involvement of additional drug transporters. A study investigating ABCG2 (BCRP) found no interaction (as either substrates or inhibitors) with major AEDs [47] and other studies have found no interaction of AEDs with ABCC1, ABCC2 and ABCC5 [48], [49]. However, an association between an ABCC2 polymorphism and carbamazepine neurological adverse reactions has been reported [50]. Although not consistent with P-gp transport (opposite direction), a decrease in carbamazepine transport by verapamil, prazosin and tariquidar in the porcine brain endothelial cells was observed. Additionally, VBL_100_ cells showed an increase in carbamazepine accumulation. This suggests an unidentified influx transporter for carbamazepine in these cells and is certainly worthy of further study. An alternative approach to trying to group AEDs with diverse physicochemical properties and structures into one interaction profile might be to consider each AED individually. For example we have recently identified lamotrigine as a substrate for the influx transporter, hOCT1, in human brain endothelial cells and this may explain its good permeability into the brain despite its unfavourable physicochemical properties [25].

In summary, P-gp does not provide a unifying basis for drug resistance in epilepsy since not all AEDs are substrates. In particular, we found no interaction of lamotrigine or carbamazepine with P-gp. Lamotrigine and carbamazepine do not conform to the properties of P-gp substrates as set out by Giacomini et al. P-gp is over-expressed in patients with refractory epilepsy [3] but its clinical relevance is not clear. Our data would suggest that P-gp does not contribute universally to the transport of AEDs in refractory epilepsy.

## Supporting Information

Figure S1 **Concentration equilibrium approach in Caco-2 monolayer.** Transport of a) 5µM ^3^H-digoxin or (b) 5µM ^3^H-digoxin in the presence of 300nM tariquidar or (c) 5µM ^14^C-phenytoin or (d) 5µM ^14^C-lamotrigine or (e) 5µM ^14^C-carbamazepine in Caco-2. Samples were taken at each indicated time point over a 4 hour time course. Data are expressed as mean ±SD (n = 3). * significantly different compared to cells without inhibitor (* P<0.05, ** P<0.01, *** P<0.001).
(TIF)

Figure S2 **Concentration equilibrium approach in LLC-PK1 transfected with human P-gp for the transport of different concentrations of lamotrigine.** Transport of a) 20µM ^14^C-lamotrigine or (b) 40µM ^14^C-lamotrigine in LLC-PK1±P-gp in the absence or presence of 300nM tariquidar. Samples were taken at each indicated time point over a 6 hour time course. Data are expressed as mean ±SD (n = 3). * significantly different compared to wild type cells. # significantly different (P<0.05) compared to LLC-PK1+P-gp cells in the absence of tariquidar.
(TIF)

Figure S3 **Concentration equilibrium approach in MDCKII transfected with human P-gp for the transport of AEDs.** Transport of a) 5µM ^3^H-digoxin or (b) 5µM ^14^C-phenytoin or (c) 5µM ^14^C-lamotrigine or (d) 5µM ^14^C-carbamazepine in MDCKII ±P-gp in the absence or presence of 300nM tariquidar. Samples were taken at each indicated time point over a 6 hour time course. * significantly different compared to wild type cells (* P<0.05, ** P<0.01, *** P<0.001.). # significantly different compared to MDCKII+P-gp cells in the absence of tariquidar (# P<0.05, ## P<0.01, ### P<0.001).
(TIF)
