# Supplementary figures and images for: A Multi-System Approach Assessing the Interaction of Anticonvulsants with P-gp
Source: PLoS One. 2013 May 31;8(5):e64854. doi: 10.1371/journal.pone.0064854 (PMC3669347; doi:10.1371/journal.pone.0064854)

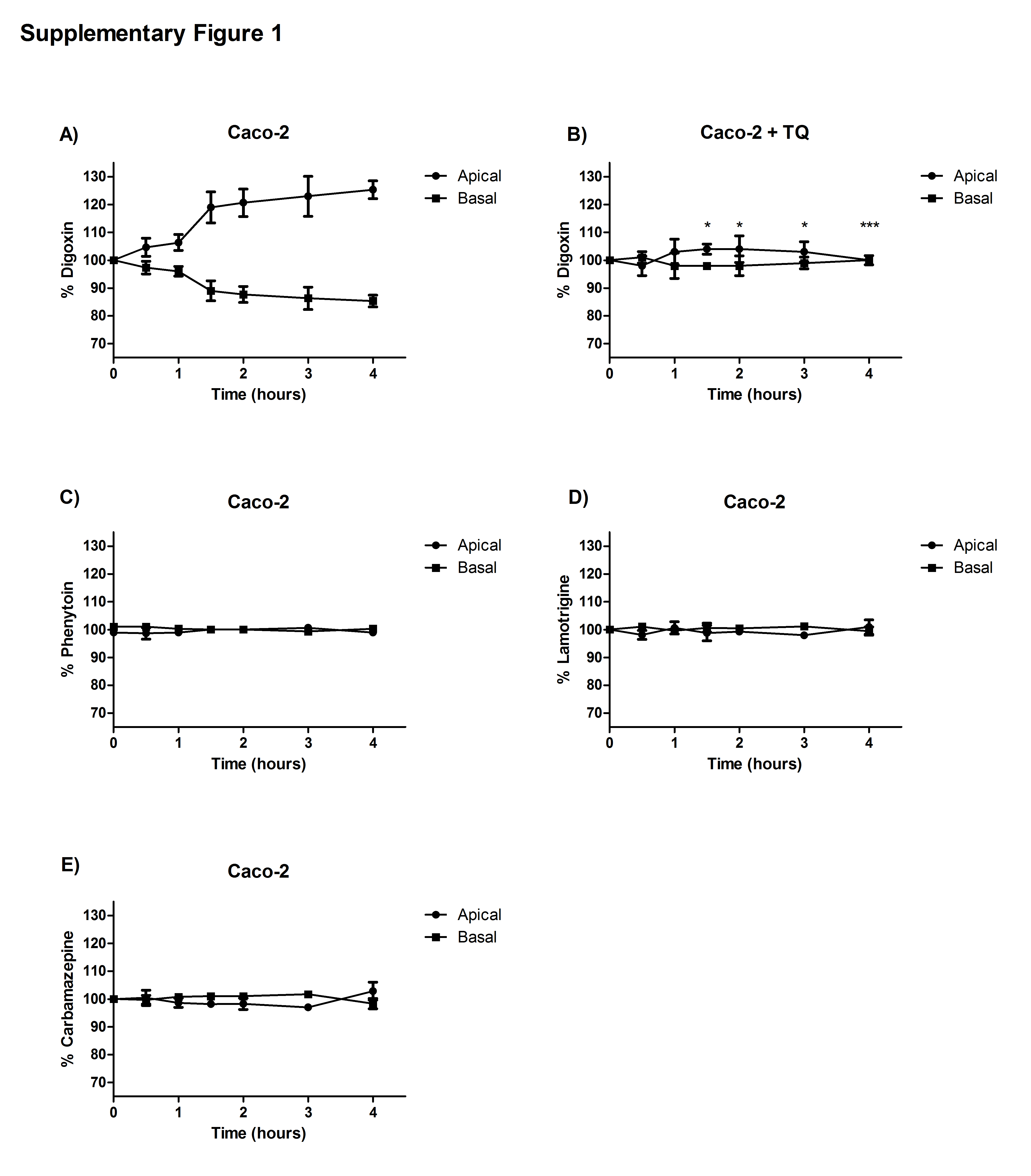

Supplement: Figure S1 — Concentration equilibrium approach in Caco-2 monolayer. Transport of a) 5µM 3H-digoxin or (b) 5µM 3H-digoxin in the presence of 300nM tariquidar or (c) 5µM 14C-phenytoin or (d) 5µM 14C-lamotrigine or (e) 5µM 14C-carbamazepine in Caco-2. Samples were taken at each indicated time point over a 4 hour time course. Data are expressed as mean ±SD (n = 3). * significantly different compared to cells without inhibitor (* P<0.05, ** P<0.01, *** P<0.001). (TIF) [file pone.0064854.s001.tif]

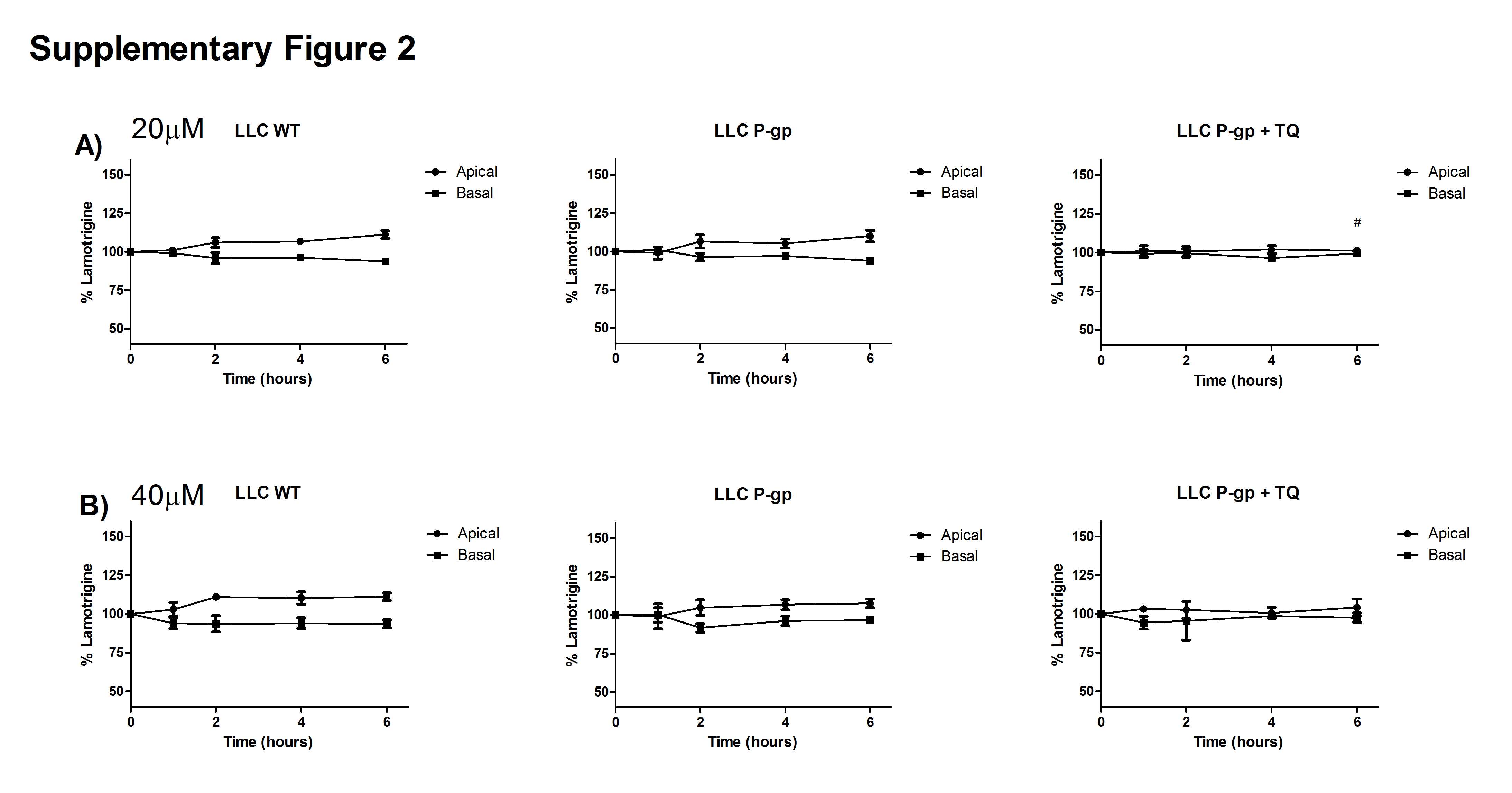

Supplement: Figure S2 — Concentration equilibrium approach in LLC-PK1 transfected with human P-gp for the transport of different concentrations of lamotrigine. Transport of a) 20µM 14C-lamotrigine or (b) 40µM 14C-lamotrigine in LLC-PK1±P-gp in the absence or presence of 300nM tariquidar. Samples were taken at each indicated time point over a 6 hour time course. Data are expressed as mean ±SD (n = 3). * significantly different compared to wild type cells. # significantly different (P<0.05) compared to LLC-PK1+P-gp cells in the absence of tariquidar. (TIF) [file pone.0064854.s002.tif]

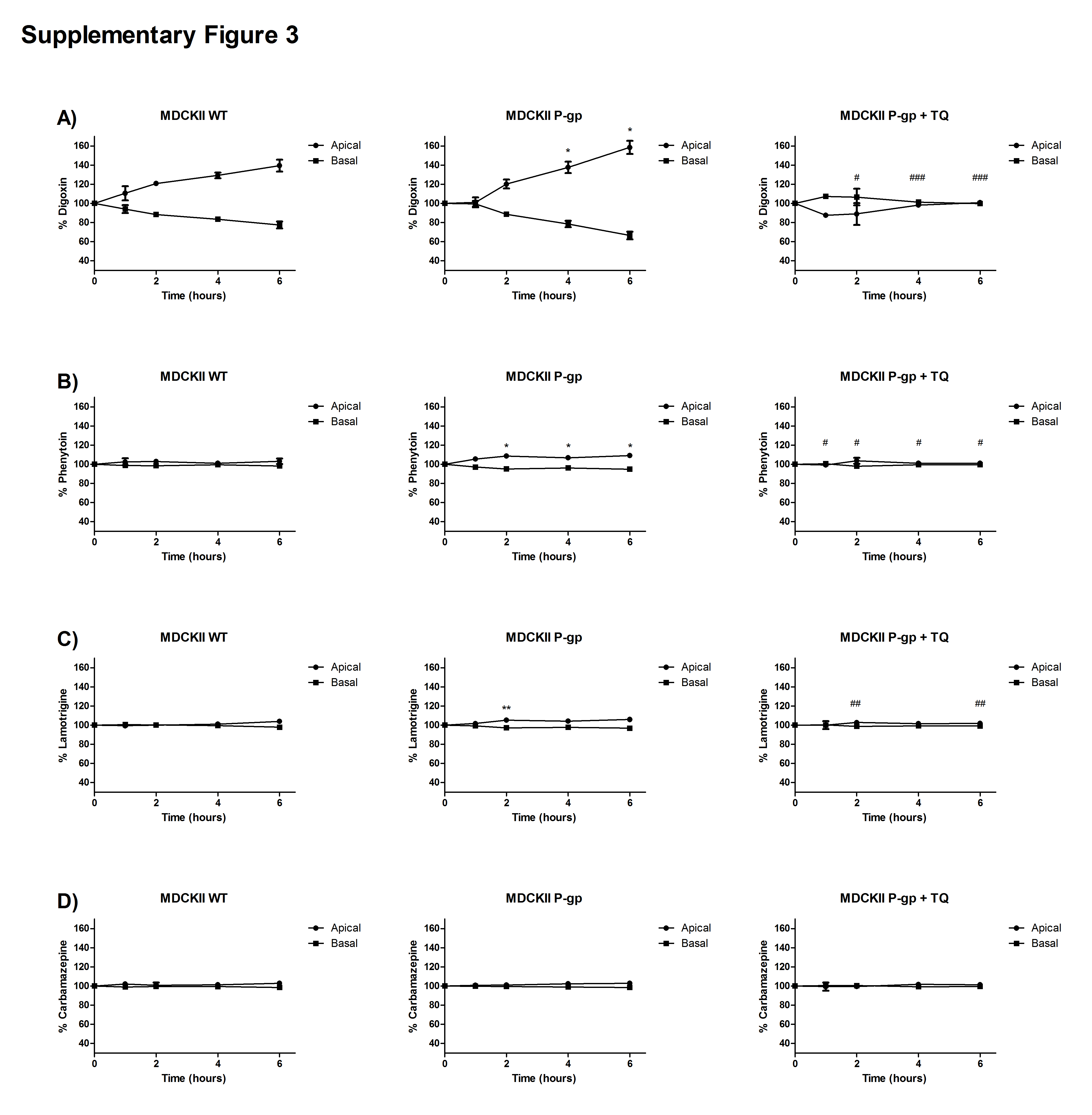

Supplement: Figure S3 — Concentration equilibrium approach in MDCKII transfected with human P-gp for the transport of AEDs. Transport of a) 5µM 3H-digoxin or (b) 5µM 14C-phenytoin or (c) 5µM 14C-lamotrigine or (d) 5µM 14C-carbamazepine in MDCKII ±P-gp in the absence or presence of 300nM tariquidar. Samples were taken at each indicated time point over a 6 hour time course. * significantly different compared to wild type cells (* P<0.05, ** P<0.01, *** P<0.001.). # significantly different compared to MDCKII+P-gp cells in the absence of tariquidar (# P<0.05, ## P<0.01, ### P<0.001). (TIF) [file pone.0064854.s003.tif]
